# Supplementary material for: HIVprotI: an integrated web based platform for prediction and design of HIV proteins inhibitors
Source: J Cheminform. 2018 Mar 9;10:12. doi: 10.1186/s13321-018-0266-y (PMC5845081; doi:10.1186/s13321-018-0266-y)
Supplement: Supplementary file 2 — Additional file 2. Source code of HIVProtI web server. [file 13321_2018_266_MOESM2_ESM.zip › HIVprotI_Source-code/hivprotI/weka-3-6-6/doc/weka/core/converters/ConverterUtils.DataSink.html]

ConverterUtils.DataSink


---


|  |  |  |
| --- | --- | --- |
| **Overview** \| **Package** \| **Class** \| **Tree** \| **Deprecated** \| **Index** \| **Help** \| **Weka's home** | |  |
| **PREV CLASS**   **NEXT CLASS** | **FRAMES**    **NO FRAMES**     **All Classes** |  |
| SUMMARY: NESTED \| FIELD \| CONSTR \| METHOD | DETAIL: FIELD \| CONSTR \| METHOD |  |


---


## weka.core.converters Class ConverterUtils.DataSink

```
java.lang.Object
  weka.core.converters.ConverterUtils.DataSink
```

**All Implemented Interfaces:**: java.io.Serializable, RevisionHandler

**Enclosing class:**: ConverterUtils

---

``` public static class ConverterUtils.DataSink extends java.lang.Object implements java.io.Serializable, RevisionHandler ```

Helper class for saving data to files. Via the ConverterUtils
class it determines which converter to use for saving the data.
It is the logical counterpart to `DataSource`.

**Version:**
:   $Revision: 6416 $

**Author:**
:   FracPete (fracpete at waikato dot ac dot nz)

**See Also:**: `ConverterUtils.DataSource`, Serialized Form

---

**Constructor Summary**

`ConverterUtils.DataSink(java.io.OutputStream stream)`
  
          initializes the sink to save the data in the stream (always in ARFF
format).

`ConverterUtils.DataSink(Saver saver)`
  
          initializes the sink to save the data to the given Saver (expected to be
fully configured).

`ConverterUtils.DataSink(java.lang.String filename)`
  
          initializes the sink to save the data to the given file.


| **Method Summary** | |
| --- | --- |
| `java.lang.String` | `getRevision()`             Returns the revision string. |
| `static void` | `main(java.lang.String[] args)`             for testing only - takes a data file as input and a data file for the output. |
| `void` | `write(Instances data)`             writes the given data either via the saver or to the defined output stream (depending on the constructor). |
| `static void` | `write(java.io.OutputStream stream, Instances data)`             writes the data to the given stream (always in ARFF format). |
| `static void` | `write(Saver saver, Instances data)`             writes the data via the given saver. |
| `static void` | `write(java.lang.String filename, Instances data)`             writes the data to the given file. |

**Methods inherited from class java.lang.Object**

`equals, getClass, hashCode, notify, notifyAll, toString, wait, wait, wait`

**Constructor Detail**

### ConverterUtils.DataSink

```
public ConverterUtils.DataSink(java.lang.String filename)
                        throws java.lang.Exception
```

:   initializes the sink to save the data to the given file.

    **Parameters:**: `filename` - the file to save data to **Throws:**: `java.lang.Exception` - if set of saver fails

---


### ConverterUtils.DataSink

```
public ConverterUtils.DataSink(Saver saver)
```

:   initializes the sink to save the data to the given Saver (expected to be
    fully configured).

    **Parameters:**: `saver` - the saver to use for saving the data

---


### ConverterUtils.DataSink

```
public ConverterUtils.DataSink(java.io.OutputStream stream)
```

:   initializes the sink to save the data in the stream (always in ARFF
    format).

    **Parameters:**: `stream` - the output stream to use for storing the data in ARFF format


**Method Detail**

### write

```
public void write(Instances data)
           throws java.lang.Exception
```

:   writes the given data either via the saver or to the defined
    output stream (depending on the constructor). In case of the stream,
    the stream is only flushed, but not closed.

    :   **Parameters:**: `data` - the data to save **Throws:**: `java.lang.Exception` - if saving fails

---


### write

```
public static void write(java.lang.String filename,
                         Instances data)
                  throws java.lang.Exception
```

:   writes the data to the given file.

    :   **Parameters:**: `filename` - the file to write the data to: `data` - the data to store **Throws:**: `java.lang.Exception` - if writing fails

---


### write

```
public static void write(Saver saver,
                         Instances data)
                  throws java.lang.Exception
```

:   writes the data via the given saver.

    :   **Parameters:**: `saver` - the saver to use for writing the data: `data` - the data to store **Throws:**: `java.lang.Exception` - if writing fails

---


### write

```
public static void write(java.io.OutputStream stream,
                         Instances data)
                  throws java.lang.Exception
```

:   writes the data to the given stream (always in ARFF format).

    :   **Parameters:**: `stream` - the stream to write the data to (ARFF format): `data` - the data to store **Throws:**: `java.lang.Exception` - if writing fails

---


### main

```
public static void main(java.lang.String[] args)
                 throws java.lang.Exception
```

:   for testing only - takes a data file as input and a data file for the
    output.

    :   **Parameters:**: `args` - the commandline arguments **Throws:**: `java.lang.Exception` - if something goes wrong

---


### getRevision

```
public java.lang.String getRevision()
```

:   Returns the revision string.

    :   **Specified by:**: `getRevision` in interface `RevisionHandler`
    :   **Returns:**: the revision


---


|  |  |  |
| --- | --- | --- |
| **Overview** \| **Package** \| **Class** \| **Tree** \| **Deprecated** \| **Index** \| **Help** \| **Weka's home** | |  |
| **PREV CLASS**   **NEXT CLASS** | **FRAMES**    **NO FRAMES**     **All Classes** |  |
| SUMMARY: NESTED \| FIELD \| CONSTR \| METHOD | DETAIL: FIELD \| CONSTR \| METHOD |  |


---
